# Transformer models in genomics: from sequence analysis to population genetics and biodiversity

**DOI:** 10.1093/bib/bbag628

**Published:** 2026-11-16

**Authors:** Iqra Farooq, Sara Atito, Inga Prokopenko, Muhammad Awais, Ayse Demirkan

**Affiliations:** Centre for Vision, Speech and Signal Processing (CVSSP), University of Surrey, Guildford, Surrey, GU2 7XH, United Kingdom; Centre for Vision, Speech and Signal Processing (CVSSP), University of Surrey, Guildford, Surrey, GU2 7XH, United Kingdom; Surrey Institute for People-Centered Artificial Intelligence (PAI), University of Surrey, Guildford, Surrey, GU2 7XH, United Kingdom; Surrey Institute for People-Centered Artificial Intelligence (PAI), University of Surrey, Guildford, Surrey, GU2 7XH, United Kingdom; Section of Statistical Multi-Omics, Department of Clinical Medicine, School of Biosciences & Medicine, University of Surrey, Guildford, Surrey, GU2 7XH, United Kingdom; Centre for Vision, Speech and Signal Processing (CVSSP), University of Surrey, Guildford, Surrey, GU2 7XH, United Kingdom; Surrey Institute for People-Centered Artificial Intelligence (PAI), University of Surrey, Guildford, Surrey, GU2 7XH, United Kingdom; Surrey Institute for People-Centered Artificial Intelligence (PAI), University of Surrey, Guildford, Surrey, GU2 7XH, United Kingdom; Section of Statistical Multi-Omics, Department of Clinical Medicine, School of Biosciences & Medicine, University of Surrey, Guildford, Surrey, GU2 7XH, United Kingdom

**Keywords:** deep learning, foundation models, genomics, attention mechanisms, sequence analysis

## Abstract

Modern genomics and artificial intelligence converge at a breakthrough point with the emergence of transformer-based large language models for genomic analysis. This review examines how transformer-based models have advanced genomic sequence analysis through novel tokenization strategies, self-attention mechanisms, and pre-training/fine-tuning paradigms. Unlike prior broad surveys, we focus on three sub-areas of genomics where transformer architectures have had a substantive impact: sequence-level analysis, population-level genetics, including genetic imputation and genome-wide association analysis, and biodiversity research, while addressing challenges like genome-scale computational demands, training biases, and interpreting attention in biological contexts. Looking forward, we outline how emerging foundation models, multi-modal integration, and biologically informed attention mechanisms could transform our understanding of genome function, providing a roadmap for leveraging genome language models to decode genome complexity and advance functional and population-level genomics.

## Introduction

Transformers are deep learning architectures that use attention mechanisms to learn relationships within sequences. They are powerful for analysing language, DNA, proteins, and other biological data because they can capture complex patterns across very large inputs. In genomics, this is especially valuable because biological meaning can depend on nearby bases, distant regulatory regions, repeated motifs, and genome-wide interactions. By identifying informative sequence regions and long-range dependencies, transformers are well suited to predicting gene regulation, interpreting non-coding variants, and detecting disease-relevant genomic patterns.

High-throughput sequencing has transformed computational genomics by generating unprecedented data volumes and enabling molecular-scale investigation of biological systems [1]. This expansion has driven increasingly sophisticated statistical and machine learning methods to extract biological insight from complex genomic datasets. Computational genomics has developed through two complementary traditions: bioinformatics methods, which analyse DNA, sequences, or structured biological information, and statistical approaches, which examine genetic variation across individuals and populations.

Sequence-based methods treat DNA, composed of A, C, G, and T, as string data, enabling nucleotide counting, transcription to RNA, alignment, motif detection, and pattern matching. These methods are often task-specific and rely on assumptions that shape the patterns they can recover. For example, position weight matrices (PWMs) represent regulatory motifs by encoding nucleotide probabilities at each position within fixed-length elements, such as transcription factor binding sites [2, 3]. Although efficient and interpretable, PWMs assume positional independence and fixed motif length, limiting their ability to capture context-dependent regulatory grammar. Profile hidden Markov models (HMMs) [4] extend this framework through match, insertion, and deletion states, supporting probabilistic alignment to models of protein domains and gene structures. They underpin tools such as HMMER [5] and Pfam [6], but their first-order Markov assumptions limit modelling of long-range sequence dependencies. Despite this, these methods have been central to genome organization, assembly, annotation, and phylogenetic comparison.

Following the Human Genome Project, genomics increasingly focused on human genetic diversity, first through clinical genetics and rare, high-impact mutations, and later through common variants with smaller effects on complex traits. Statistical genetics recodes variable genomic positions into numerical or binary representations for population-scale analysis, enabling biobank studies and personalized medicine frameworks. Key methods include genome-wide association studies (GWAS), polygenic risk score prediction, and classification approaches across human, animal, and plant populations. At population scale, GWAS test millions of single-nucleotide polymorphisms for association with phenotypes [7–9]. Standard approaches fit separate linear or logistic regression models at each variant, adjusting for population structure using principal components, while mixed-model methods such as BOLT-LMM [10] and SAIGE [11] account for cryptic relatedness. Although GWAS has identified many trait-associated loci, stringent significance thresholds, typically $p<5 \times 10^{-8}$, and multiple-testing correction [12] may discard informative sub-threshold signals. Moreover, standard GWAS evaluates variants largely independently and does not directly model epistasis [13, 14], non-additive effects, or surrounding sequence context. These limitations across motif models, HMMs, and GWAS created methodological gaps that machine learning approaches have sought to address [15]. Among them, convolutional neural networks (CNNs) gained atttraction in genomics around 2015, with DeepBind [16] and DeepSEA [17] demonstrating performance gains in predicting regulatory elements by identifying local sequence motifs [18]. Recurrent neural networks (RNNs), particularly long short-term memory architectures, were adapted to capture longer-range dependencies [19, 20]. Models like DanQ [21] combined CNNs and RNNs to hierarchically extract both local and distal patterns. However, CNNs struggled with distant sequence dependencies critical for gene regulation [22], while RNNs faced vanishing gradient problems and computational inefficiencies [23, 24].

The transformer architecture [25], introduced in 2017, marked a paradigm shift by eschewing recurrence and convolution entirely, relying instead on self-attention mechanisms to model relationships between any sequence positions regardless of distance while maintaining computational efficiency. The success of BERT [26], GPT [27], and T5 [28] in natural language processing suggested potential for analysing and understanding biological sequences.

Parallels between natural language and genomic sequences provide a compelling rationale for applying transformers to genomic data. Both are structured, sequential data with complex grammar encoding functional information, exhibiting hierarchical organization where smaller units combine to form larger functional elements with context-dependent meanings. Most importantly, both feature critical long-range dependencies determining functional outcomes. In language, distant word relationships alter meaning, while in genomics, distal regulatory element interactions profoundly influence gene expression. The same parallel has motivated transformer language models across other biological sequence types, including proteome-scale analysis [29].

Beginning around 2020, researchers began developing transformer-based models specifically adapted for genomic analysis. The pioneering DNABERT [30] model applied the BERT framework to genomic sequences, demonstrating performance in predicting various DNA functional elements. Subsequent models such as DNABERT-2 [31], Nucleotide Transformer [32], and GENA-LM [33] introduced innovations in tokenization, attention mechanisms, and architectural design, achieving state-of-the-art performance across regulatory element identification, variant effect prediction, and functional annotation. Transformer–convolution hybrids have also been applied to base-resolution tasks such as identifying DNA N6-methyladenine sites across species [34].

While transformer-based approaches have gained significant traction in sequence-level genomic analysis, their application to population-level genetic studies, particularly GWAS, remains limited. Traditional machine learning approaches, including support vector machines, random forests, and deep neural networks, have been adapted for GWAS [35–39]. These approaches aim to overcome limitations of conventional statistical methods by better modelling complex genetic architectures and interactions. However, there exists a notable methodological gap between sequence-focused transformer models and population-level machine learning approaches, representing an important opportunity for future research and development.

Several recent reviews survey transformer and language model approaches in genomics: Zhang *et al*. [40] cover transformer-based language models broadly across bioinformatics; Consens *et al*. [41] and Benegas *et al*. [42] examine genome language models with a focus on architectural design and opportunities for sequence-level analysis; Ghosh *et al*. [43] survey transformers for nucleotide sequence analysis; Choi and Lee [44] review transformer architectures and attention mechanisms for genome data; and Shu *et al*. [45] provide a comprehensive survey of genome language models in bioinformatics. These reviews are valuable references for the foundation-model literature, and we draw on them throughout.

Our contribution differs in three ways. First, we connect sequence-level foundation models to the population-level transformer literature within a single comparative frame, including imputation (HLARIMNT, STICI), GWAS-pipeline applications (SNVformer, InsightGWAS, ViT-AE GWAS), end-to-end genotype-to-phenotype models (Phenformer, COMICAL), and biodiversity (BarcodeBERT); areas treated only briefly or omitted in the prior reviews. Second, our comparative analysis (section Comparative analysis across models) is organized by axes of trade-off (context length versus granularity, training paradigm and transferability, cross-population and cross-species generalization) rather than by per-model specifications, foregrounding what is and is not yet comparable. Third, we are explicit about where the field is methodologically unsettled, e.g. that transformer-based GWAS is not yet a single methodology but a collection of methods that intersect the GWAS pipeline at different stages. Figure 1 maps the models reviewed here onto these subfields and onto the architectural timeline of the field.

**Figure 1:**
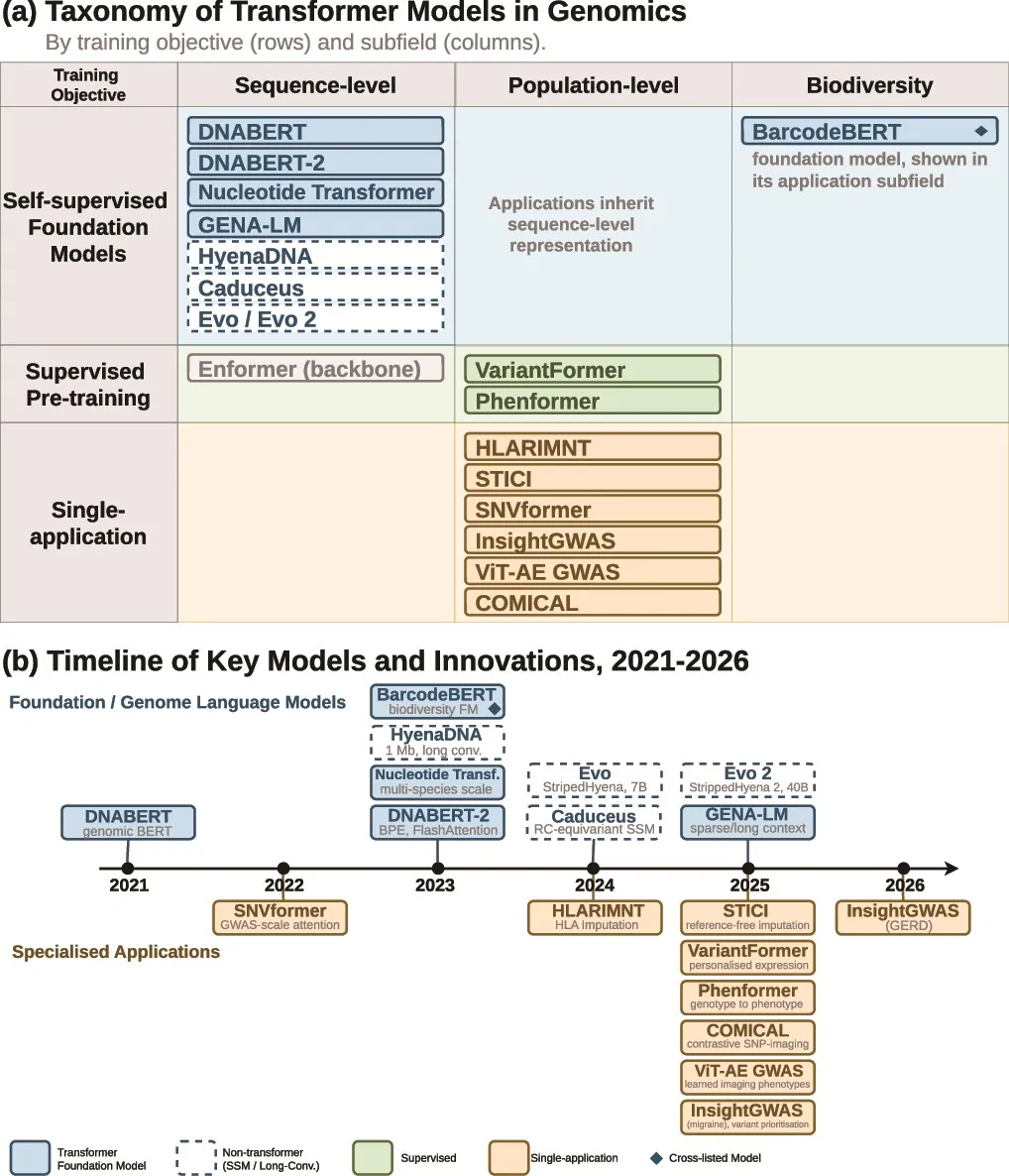
Overview of transformer and related genome models reviewed. (a) Taxonomy organized by training objective (rows: self-supervised foundation models, supervised pre-training, and single-application models) and by subfield (columns: sequence-level, population-level, and biodiversity). The dashed outlines denote non-transformer long-context or state-space genome language models (HyenaDNA, Caduceus, and the Evo family); the diamond badge marks BarcodeBERT, a foundation model discussed within the biodiversity subfield. Empty population- and biodiversity-level foundation cells reflect that these subfields are populated chiefly by models that inherit sequence-level representations rather than by dedicated foundation models. (b) Timeline of representative models, 2021–6, with foundation and genome language models above the axis (annotated with each model’s principal innovation) and specialized applications below, showing both the architectural progression from k-mer encoders to long-context and SSMs and the later emergence of population-level and biodiversity applications.

We focus on three subfields deliberately rather than exhaustively, selecting the areas in which transformer architectures have produced distinct methodological contributions rather than incremental re-application. Sequence-level analysis is where genomic transformers originated and where they are most mature, providing the foundation-model representations on which later work builds. Population-level genetics, including genotype imputation, genome-wide association, and genotype-to-phenotype prediction, is a rapidly growing area of personalized medicine and it is also where the integration of transformers is least settled: methods enter the analysis pipeline at different stages, report incommensurable metrics, and coexist with well-established statistical approaches, making a comparative treatment particularly useful. Biodiversity is the clearest test of transfer beyond human and model-organism genomes, and therefore the sharpest probe of the generalization claims made for foundation models. These three areas are also connected by a single thread that motivates treating them together: representations learned at the sequence level are inherited, together with their biases, by population-level and cross-species applications, so that the same limits on generalization recur across all three (Section Cross-population and cross-species generalization). We do not cover areas such as single-cell or protein-structure transformers, which are the subject of dedicated reviews [40, 45].

This review examines current transformer-based genomic models, covering self-supervised learning (SSL), transformer architectures, genomic tokenization, and pre-training strategies. We compare model designs and benchmarks, discuss challenges in scalability, data quality, interpretability, and ethics, and highlight future directions including multimodal integration, biological knowledge incorporation, computational efficiency, and clinical validation. The review provides practical insights for applying transformers to genomic discovery.

**A note on terminology.** We use *transformer models* throughout to refer to architectures built on the self-attention mechanism of Vaswani *et al*. [25], regardless of training objective. *Genome language models* is a narrower category covering transformers (and some non-transformer architectures, e.g. HyenaDNA [46] based on long convolutions) trained with self-supervised sequence modelling objectives such as masked or autoregressive prediction. *Foundation models* are the subset of genome language models pre-trained at scale on large unlabelled corpora and intended for adaptation to many downstream tasks via fine-tuning. The categories overlap but are not identical: Enformer is a transformer but not a genome language model in this sense (it is trained supervisedly on functional genomics tracks); HyenaDNA is a genome language model but not a transformer; DNABERT is all three. Where the distinction matters in this review, we name the relevant category explicitly.

Section Background and basic concepts establishes the architectural and SSL foundations that the remainder of the review builds on. A glossary of machine learning and genomics terms used throughout this review is provided in the supplementary material for readers from adjacent fields.

## Background and basic concepts

Understanding transformer-based genomic language models requires grounding in SSL paradigms that enable extraction of meaningful representations from vast unlabelled data. This section provides essential foundations for readers from diverse backgrounds, covering self-supervised learning principles, transformer architecture, and genomic-specific adaptations.

### Self-supervised learning foundations

Transformer-based genomic models rely on SSL, in which supervisory signals are derived from the input sequences themselves rather than from manual labels. Two SSL objectives dominate the literature: masked language modelling, used by BERT-style encoders, and autoregressive modelling, used by GPT-style decoders (see Supplementary Material for definitions). Combined with the standard pre-training and fine-tuning paradigm, SSL allows models to learn general-purpose representations from large unlabelled corpora before being adapted to specific downstream tasks with limited labelled data [47–51]. This approach has become foundational in natural language processing and underpins all genomic transformer models discussed in this review.

### Transformer architecture

The transformer architecture [25] represents a fundamental departure from recurrent or convolutional approaches, built around self-attention mechanisms allowing models to consider all sequence positions when encoding any given position.

The standard transformer consists of an encoder–decoder structure (Fig. 2a). The encoder maps input sequences to continuous representations, which the decoder uses to generate output sequences. Each encoder layer contains a multi-head self-attention and a position-wise feed-forward network. The decoder has a similar structure plus an additional attention to the encoder outputs (Fig. 2b).

**Figure 2:**
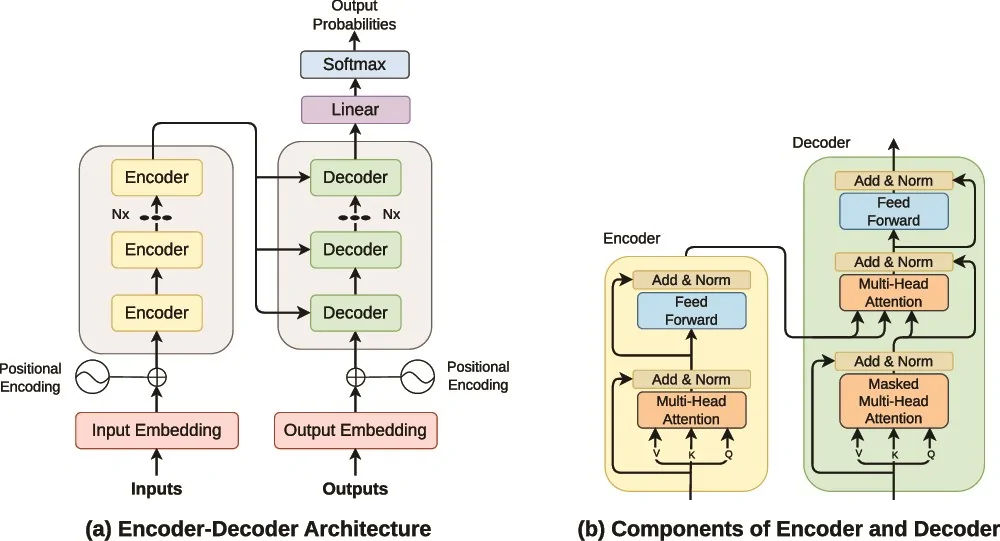
Transformer architecture: (a) Overall transformer architecture showing the encoder–decoder structure, and (b) Detailed internal structure of transformer encoder and decoder layers. Each layer consists of multi-head attention mechanisms, position-wise feed-forward networks (Feed Forward), and residual connections combined with layer normalization (denoted **Add & Norm**). **QK**, and **V** denote the query, key, and value vectors used as inputs to the attention mechanism. The decoder additionally includes masked multi-head attention to prevent attending to future positions. **N*×*** indicates that the encoder and decoder block is stacked N times.

**Self-attention mechanism.** For an input sequence, $X\in \mathbb{R}^{n\times d_{\mathrm{model}}}$ with *n* tokens and embedding dimension $d_{\mathrm{model}}$, query (*Q*), key (*K*), and value (*V*) vectors are computed as


(1) \begin{eqnarray*}& Q=XW^{Q}, \quad K=XW^{K}, \quad V=XW^{V},\end{eqnarray*}

where $W^{Q},W^{K}\in \mathbb{R}^{d_{\mathrm{model}}\times d_{k}}$, and $W^{V}\in \mathbb{R}^{d_{\mathrm{model}}\times d_{v}}$ are learnable parameters.

Attention weights between positions are calculated as scaled dot products of query and key vectors, followed by softmax normalization, which converts the raw similarity scores into a probability distribution over input positions. These weights then compute weighted sums of value vectors, producing context-aware representations (Fig. 3a):


(2) \begin{eqnarray*}& \mathrm{Attention}(Q,K,V)=\mathrm{softmax}\left(\frac{QK^{T}}{\sqrt{d_{K}}}\right)V\end{eqnarray*}

**Figure 3:**
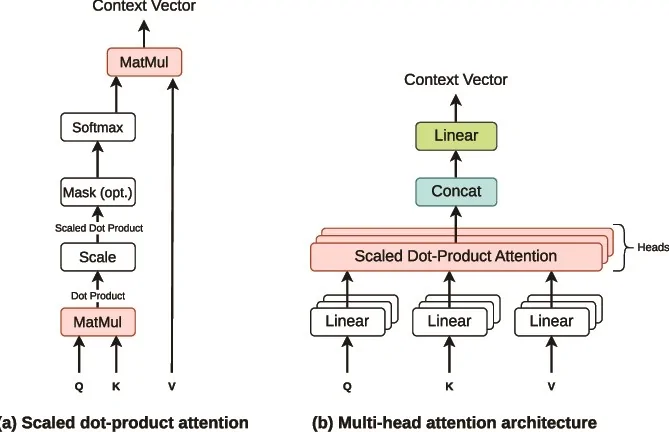
Attention mechanisms in transformers: (a) Scaled dot-product attention computation flow. The mechanism takes query (**Q**), key (**K**), and value (**V**) matrices as input. **MatMul** denotes matrix multiplication; **Scale** denotes division by $\sqrt{d_{k}}$, where $d_{k}$ is the dimension of the key vectors. **Mask (opt.)** is an optional masking step used in decoder self-attention to prevent attending to future positions. **Softmax** normalizes the resulting scores into attention weights (a probability distribution over input positions). The final **MatMul** combines these weights with the value matrix to produce the context vector. **(b)** Multi-head attention mechanism showing how multiple attention heads process the input in parallel. **Linear** denotes a learned linear projection. Each head applies its own projections to Q, K, and V; performs scaled dot-product attention independently, and the resulting context vectors are concatenated (**Concat**) and passed through a final linear projection to produce the output.

The scaling factor $\sqrt{d_{k}}$ prevents large dot products that would push softmax into regions with small gradients.

**Multi-head attention** extends by allowing the model to jointly attend to information from different representation subspaces. This is achieved by linearly projecting queries, keys, and values *h* times with different learned projections, performing attention in parallel, then concatenating and projecting the results (Fig. 3b).


(3) \begin{eqnarray*}& \mathrm{MultiHead}(Q,K,V)=\mathrm{Concat}(\mathrm{head}_{1},\ldots,\mathrm{head}_{h})W^{O},\end{eqnarray*}

where each head applies its own learned projections to *Q*, *K*, and *V* before attention, and $W^{O}$ projects the concatenated heads back to the model dimension; typically $d_{k} = d_{v} = d_{\mathrm{model}}/h$, so that multi-head attention retains a computational cost similar to single-head attention at full dimensionality.

**Positional encoding**. To preserve token position information, transformers incorporate positional encodings added to input embeddings. The original transformer used sinusoidal encodings, though subsequent models have explored alternative approaches such as learned positional embeddings or relative positional encodings.

Each attention sub-layer is followed by a position-wise feed-forward network, with residual connections and layer normalization applied around both sub-layers; the encoder stacks *N* such layers, and the decoder adds masked self-attention and cross-attention to the encoder output.

### Adaptation for genomic data

Having established the transformer architecture’s core components, we now examine how these principles must be adapted to address the unique characteristics of genomic sequences. Genomic sequences present unique challenges: consisting of only four nucleotides (A,T,G,C) yet spanning millions to billions of base pairs with critical functional elements dispersed across vast non-coding regions [52]. This presents a fundamental challenge: the self-attention mechanism’s quadratic complexity becomes computationally prohibitive for genome-scale analysis, while limited alphabet size risks underutilizing representational capacity.

Addressing these constraints requires specialized tokenization strategies that generate the token embeddings fed into the input layer described in section Transformer architecture. Similarly, pre-training objectives must adapt the masked language modelling framework from section Self-supervised learning foundations to capture biological sequence dependencies and evolutionary constraints. The following subsections detail these genomic-specific tokenization approaches and pre-training strategies.

#### Tokenization strategies

The choice of tokenization strategy fundamentally shapes a transformer model’s ability to capture both local sequence patterns and distant regulatory relationships, and represents one of the most active areas of design in genomic language modelling. Figure 4 illustrates the principal approaches now in use.

**Figure 4:**
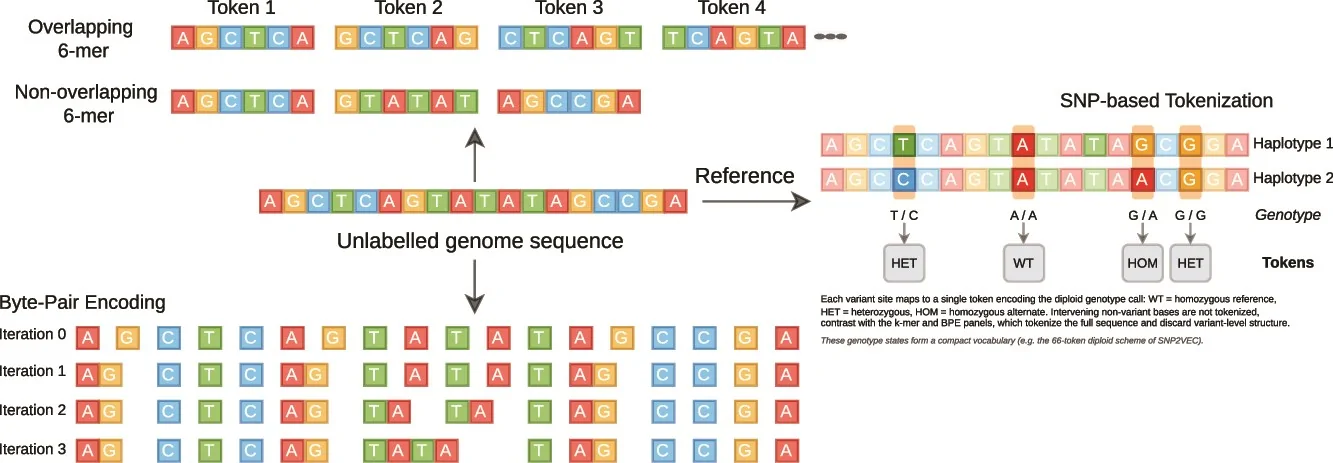
Comparison of genomic sequence tokenization strategies. The top panels illustrate overlapping versus non-overlapping 6-mer tokenization of a DNA sequence, showing how different approaches segment the same genomic input. The BPE panel demonstrates the iterative process by which frequent nucleotide pairs are progressively merged across multiple iterations to build a vocabulary of biologically relevant patterns. The SNP-based panel illustrates the population-genetics approach, in which each variant site is mapped to a single token encoding the diploid genotype call (WT, homozygous reference; HET, heterozygous; HOM, homozygous alternate); intervening non-variant bases are not tokenized, so surrounding sequence context is discarded in exchange for population-level alignment. Such genotype states form a compact vocabulary, e.g. the 66-token diploid scheme of SNP2Vec.

**K-mer tokenization** divides sequences into subsequences of length *k*, expanding vocabulary from 4 nucleotides to $4^{k}$ possible k-mers. This captures local motifs corresponding to functional elements such as transcription factor binding sites. Tokenization can be overlapping, in which a sliding window of stride one yields one token per nucleotide and explicitly encodes positional context, or non-overlapping, in which the sequence is partitioned into disjoint k-mers, producing fewer tokens at the cost of fixed segmentation boundaries. DNABERT [30] uses overlapping k-mer tokenization with k = 3 to 6, while Nucleotide Transformer [32] uses non-overlapping 6-mers. K-mer schemes are simple and biologically interpretable, but they conflate compression with vocabulary explosion and can cause information leakage between adjacent tokens in the overlapping variant.

**Byte pair encoding (BPE)** adapted from natural language processing [53] iteratively merges the most frequent adjacent token pairs to build a variable-length vocabulary. This data-driven approach allows recurrent biological motifs (promoter elements, repeat units, regulatory grammars) to emerge as single tokens without prior biological annotation. DNABERT-2 [31] and GENA-LM [33] both employ BPE, which yields shorter token sequences for the same input length and removes the rigidity of a fixed k.

**SNP-based tokenization** departs from sequence-level approaches entirely by treating each genetic variant as the atomic unit. This is the approach taken by population-genetics transformers, including HLARIMNT [54], STICI [55], and SNVformer [56], where the input is a sequence of SNPs rather than a stretch of nucleotides. SNP2Vec [57] develops this direction further by encoding diploid SNP variation explicitly: rather than collapsing genotypes onto a haploid reference sequence, it builds a 66-token vocabulary covering wild-type, heterozygous, and homozygous variations on each chromosome, enabling self-supervised pre-training on diploid genomes.

The trade-offs across these strategies are summarized by three competing pressures: *resolution* (single-nucleotide schemes preserve every base but inflate sequence length), *compression* (BPE and non-overlapping k-mers reduce sequence length but obscure base-level signal), and *biological alignment* (SNP-based schemes are natural for population genetics but discard the surrounding sequence context). No single approach dominates across all genomic tasks, and the right tokenizer depends on whether the model is intended to capture sequence-level grammar, variant-level effects, or population-level structure.

#### Pre-training and fine-tuning

Genomic transformers such as DNABERT, DNABERT-2, Nucleotide Transformer follow the standard two-stage pre-training and fine-tuning paradigm, but the genomic context shapes both stages in specific ways. Masked language modelling on raw genome sequence implicitly forces the model to learn three types of structure that are otherwise expensive to supervise. First, *base composition and motif structure:* predicting a masked nucleotide or k-mer requires capturing local sequence regularities such as transcription factor binding motifs, splice donor and acceptor signals, and CpG patterns, since these are the contexts in which masked positions are most predictable. Second, *evolutionary constraint:* when pre-training is performed across multi-species genomes, the model is rewarded for learning sequence preferences that are conserved across taxa, which serve as a proxy for functional importance and reduce the need for explicit conservation features at fine-tuning time [58]. Third, *long-range dependency structure:* even within a masked-token objective, predicting a masked position from a distant context teaches the model that regulatory elements interact across kilobases.

Compared with purely supervised modelling, pre-training has two practical advantages and two costs. The advantages are sample efficiency on downstream tasks, since fine-tuning requires labelled data on the order of hundreds to thousands of examples rather than the hundreds of thousands typical of from-scratch supervised models [59], and breadth of transfer, since a single pre-trained backbone can be fine-tuned for promoter prediction, splice-site classification, variant effect, and chromatin accessibility without architectural changes. The costs are large compute requirements at the pre-training stage and a tendency for the learned representations to reflect the composition of the pre-training corpus, which in practice means a bias towards European-ancestry references and well-studied model organisms (Section Data quality and population representation). Whether pre-training improves over supervised baselines also depends on the downstream task: tasks with abundant labelled data and short input contexts (e.g. some classical motif prediction benchmarks) gain less from pre-training than tasks with sparse labels or long-range dependencies (e.g. distal regulatory element prediction). The remainder of this review examines how the foundation models in Section Genomic foundation models, the application-specific transformers in Section Specialized transformer applications, and the comparative evaluation in Section Comparative analysis across models instantiate these design choices in practice.

## Transformer models for genomic analysis

We organize the reviewed models along two axes. The first is **training objective:** following the Introduction, a *foundation model* is pre-trained with a self-supervised objective (masked or autoregressive sequence modelling) at scale on unlabelled corpora and adapted to many downstream tasks, whereas a *specialized application* is trained, with or without supervised pre-training, for a single task such as imputation, association analysis, or species classification. We apply this criterion consistently in the text and supplementary tables. Two cases are worth flagging. VariantFormer extends foundation-model ideas but is trained supervisedly on paired sequence and expression data, and is therefore treated as a specialized genotype-to-phenotype model (Section GWAS and genotype-to-phenotype applications, Table S2). BarcodeBERT, conversely, is a self-supervised foundation model (Table S1) that we nonetheless discuss under biodiversity (Section Biodiversity applications), where its significance, namely transfer to non-model taxa, lies; it is the review’s one deliberate cross-listing, flagged *in situ*.

The second axis is **subfield**. Section Genomic foundation models covers the foundation models underpinning sequence-level analysis, together with the long-context and state-space architectures now extending the accessible sequence range. Section Specialized transformer applications covers the specialized applications populating the population-genetics subfield (imputation, 5; GWAS and genotype-to-phenotype modelling, 5) and biodiversity (5). Across both axes, one observation unifies the review: population-level and cross-species applications inherit the representations, and the ancestry and species biases, of the sequence-level models they build on, so the generalization limits analysed in Section Cross-population and cross-species generalization are common to all three subfields rather than specific to any one. Figure 1 presents this organization as a taxonomy and timeline.

### Genomic foundation models

**DNABERT and DNABERT-2**

DNABERT [30] pioneered the application of BERT to genomic DNA analysis, targeting regulatory element prediction, including promoters, transcription factor binding sites, and splice sites. It uses overlapping 6-mer tokenization and masked language modelling, where *∼*15% of tokens are masked during pre-training and predicted from bidirectional sequence context. Pre-trained on the human reference genome hg38, DNABERT demonstrated utility across promoter prediction, splice site detection, and transcription factor binding site prediction. The architecture consists of multiple transformer encoder layers with multi-head self-attention and feed-forward networks, allowing simultaneous attention to different sequence context aspects. The pre-trained model fine-tunes on specific tasks with relatively small labelled datasets, limited to 512 base pair sequences.

DNABERT-2 [31] introduced significant enhancements: replacing k-mer tokenization with BPE, incorporating Attention with Linear Biases (ALiBi) for improved positional encoding, implementing FlashAttention, and using Low Precision Layer Normalization for greater flexibility and memory efficiency. Training included multi-species genomes, enhancing cross-organism generalizability, additional predicting transcription factor binding sites (human), epigenetic marks (mouse), and COVID variant classification (virus). DNABERT-2 outperformed its predecessor across most tasks in the Genome Understanding Evaluation (GUE) benchmark, a suite of 28 datasets covering core regulatory element identification tasks with notable improvements in cross-species generalization and computational efficiency. Its ability to handle longer input sequences (up to 10 000 nucleotides compared with DNABERT’s 512) expanded applicability.

**Nucleotide Transformer**

The Nucleotide Transformer [32] is an encoder-only genomic foundation model using 6-mer tokenization for tasks such as regulatory element prediction, epigenetic mark inference, splice site detection, and variant effect prediction. It was pre-trained on human reference data, 3202 genomes from the 1000 Genomes Project and 850 diverse non-human genomes, using masked language modelling and sequence chunking to capture local and broader genomic context. Nucleotide Transformer demonstrated versatility across multiple tasks: achieving state-of-the-art performance in predicting enhancers, promoters, and regulatory regions across cell types; accurately predicting histone modifications and DNA methylation patterns from sequence alone; effectively identifying exon–intron boundaries; and showing promise in predicting the functional impact of human genetic variants, e.g. in classifying ClinVar pathogenic variants

**GENA-LM**

GENA-LM [33] addresses a central challenge in applying transformers to genomic data: handling long sequences. With three distinct variants employing different attention mechanisms, GENA-LM offers flexibility for varying genomic sequence lengths. GENA-LM employs BPE similar to DNABERT-2, enabling efficient sequence representation. Its tripartite approach includes Foundational GENA Models utilizing conventional full attention for sequences up to 4.5 kb, providing comprehensive dependency capture between all positions; Sparse GENA Models, which adapt the BigBird [60] sparse attention pattern, combining local windowed attention, global tokens, and random connections to maintain long-range relationship modelling while reducing the quadratic cost of full attention that enables sequences up to 36 kb without sacrificing the ability to capture distal dependencies; and Recurrent GENA Models incorporating memory capabilities to process extremely long sequences through segmentation, maintaining memory of previous segments to theoretically process unlimited-length sequences, suitable for entire chromosomes or genomes.

Pre-training used the human T2T v2 reference genome, the 1000 Genomes Project, and a multi-species dataset from ENSEMBL release 106. Downstream evaluations covered regulatory element activity prediction, splice site detection, chromatin profiling, and polyadenylation site usage. GENA-LM variants achieved competitive performance across these tasks, with the Sparse and Recurrent variants showing the largest improvements on tasks requiring context beyond 2 kb.

The principal contribution of GENA-LM is sequence-length scalability across three configurations: Foundational models provide full attention capacity for short regulatory elements; Sparse and Recurrent variants extend tractable input length to 36 kb and beyond, enabling tasks such as enhancer-promoter interactions and topologically associating domain analysis. This addresses the quadratic scaling of standard self-attention without abandoning the architecture’s representational properties.

Read together, the foundation models trace a single trajectory driven by one constraint: the tension between resolution and context length. DNABERT established that masked-language pre-training on raw sequence yields transferable representations, but its overlapping k-mer tokenization and 512-bp window confined it to local tasks. DNABERT-2 and the Nucleotide Transformer relaxed the vocabulary and length limits through BPE and longer windows; GENA-LM relaxed the length limit further through sparse attention; and the long-context and state-space models (SSM) of Section Beyond attention: long-context and state-space models removed it almost entirely. What has not advanced at the same rate is evidence that longer context is consistently exploited: as Section Context length versus granularity shows, added context helps some tasks and not others. The most important open question for this class of model is therefore no longer how to reach genome-scale context, but when that context carries signal a shorter model would miss.

#### Beyond attention: long-context and state-space models

The quadratic cost of self-attention has motivated a parallel line of genomic sequence models that replace or supplement attention with sub-quadratic operators, extending the tractable context by orders of magnitude. These models are genome language models in the sense used here, meaning self-supervised sequence models, but most are not transformers, and we include them because they define the long-context frontier against which transformer models are increasingly measured.

**HyenaDNA** [46] replaces attention with the Hyena operator, a combination of implicit long convolutions and data-controlled gating, and operates as a decoder-only autoregressive model at single-nucleotide resolution. Pre-trained on the human reference genome, it scales to context lengths of up to one million tokens, roughly a 500-fold increase over dense-attention models of its time, while retaining single-base resolution, and it is competitive with Nucleotide Transformer and DNABERT-2 on several benchmarks despite far fewer parameters.

**State-space models** offer an alternative route to linear-time sequence modelling. Mamba [61] introduced a selective state-space mechanism that propagates a compressed hidden state along the sequence, achieving near-linear scaling in length. Because a causal, unidirectional state is ill-suited to DNA, in which both strands and both flanking directions carry information, Caduceus [62] adapts Mamba for genomics through a bi-directional block (BiMamba) and a reverse-complement-equivariant block (MambaDNA), yielding the first family of RC-equivariant bi-directional long-range DNA language models. Trained on sequences up to 131 kb, Caduceus reported stronger downstream performance than HyenaDNA and several transformer baselines, and on a long-range variant-effect task it exceeded models an order of magnitude larger that lack bi-directionality and equivariance, evidence that architectural inductive biases matched to DNA structure can substitute for scale. The same idea has since been carried into biodiversity, where an SSM barcode model has been proposed as a successor to k-mer transformer barcode encoders [63].

**The Evo family** scales the hybrid approach to genome length. Evo [64] uses the StripedHyena architecture, which interleaves long convolutions with a minority of attention layers, with seven billion parameters, pre-trained autoregressively on prokaryotic and phage genomes and extended to 131 kb context. Evo 2 [65] extends this to models of seven and forty billion parameters with a one-million-base-pair context window at single-nucleotide resolution, trained on roughly 9.3 trillion base pairs spanning all domains of life using the StripedHyena 2 architecture, and predicts the functional effects of variation, including clinically significant coding variants, from sequence alone without task-specific fine-tuning. Evo 2 illustrates both the promise and the cost of the frontier: genome-scale context and cross-domain generalization on one hand, and training budgets far beyond the reach of individual laboratories on the other (Section Computational scalability).

For readers, the practical significance of this line is that context length is no longer the binding constraint it was for the encoder-only transformers of Section Genomic foundation models; the open questions have shifted to whether long context is usefully exploited on a given task, and to the reproducibility and accessibility costs of models at this scale (Sections Context length versus granularity, Computational scalability, Benchmarking and reproducibility).

### Specialized transformer applications

#### Genetic imputation models

Genetic imputation is a critical preprocessing step in population-scale genomics, increasing the variant coverage of genotyped cohorts by leveraging reference haplotype panels. Transformer-based approaches have recently advanced this field, offering improved accuracy over traditional statistical methods (Fig. 5).

**Figure 5:**
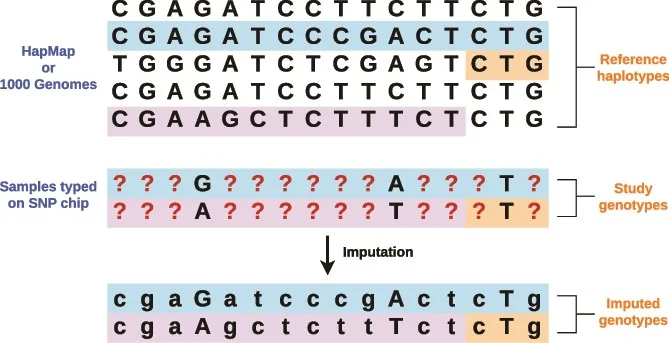
Genetic imputation workflow. Imputation uses reference haplotype panels (top) to infer missing genotypes (?) in study samples (middle), producing complete genotype data (bottom) by leveraging linkage disequilibrium patterns.

**HLARIMNT** [54] addresses HLA allele imputation from pre-phased SNP data, building on the architecture of the earlier CNN-based DEEP*HLA but replacing convolutions with a transformer encoder. SNP haplotypes are encoded as binary vectors, divided into chunks within an embedding layer, and processed by transformer layers with self-attention to model dependencies across the major histocompatibility complex region. A classification head outputs allele dosages for eight classical HLA genes at four-digit resolution.

The model is evaluated on three reference panels: the Pan-Asian panel, the T1DGC panel, and a mixed panel constructed from 530 Pan-Asian and 530 randomly selected T1DGC individuals. HLARIMNT achieves higher accuracy than DEEP*HLA across most genes and evaluation indices ($r^{2}$, PPV, sensitivity, and probability), with the largest gains on infrequent alleles (frequency below 1%) where deep learning methods typically struggle. Performance also remained higher than DEEP*HLA across a range of training-set sizes. The mixed-panel evaluation shows that HLARIMNT can be trained on ancestrally heterogeneous data without overfitting to one ancestry group, although the paper does not specifically benchmark performance on populations underrepresented in either source panel.

**STICI (Split-Transformer with Integrated Convolutions for Imputation)** [55] is a reference-free genotype imputation model for both single-nucleotide and structural variants. It combines convolutional blocks to capture local linkage-disequilibrium patterns with transformer self-attention to model long-range genotype correlations. Trained using self-supervised genotype masking and reconstruction, STICI can learn directly from genome collections and be applied to new target datasets without retraining.

Evaluated on human, simulated and non-human genomes, STICI achieved SNV imputation accuracy comparable with classical methods such as Beagle5.4, Minimac4, Impute5, and SHAPEIT5, while also supporting multi-allelic and structural variants where many tools are limited. Its strongest performance occurred in regions with tightly linked variants, suggesting effective learning of linkage-disequilibrium patterns. Cross-species results showed generalizability beyond human data, although underrepresented human ancestries were not specifically benchmarked.

HLARIMNT and STICI represent complementary strategies, locus-specialized versus general and reference-free, and both improve on strong classical baselines in their respective settings. Neither, however, evaluates the setting that matters most for equitable deployment: imputation accuracy in ancestrally under-represented populations. This gap, shared with the foundation models (Section Data quality and population representation), is the principal barrier to using transformer imputation in the diverse cohorts that stand to benefit most.

#### GWAS and genotype-to-phenotype applications

Transformer-based methods intersect with the GWAS workflow at three different layers: as direct phenotype classifiers from genotype data, as post-GWAS variant-prioritization models that re-rank summary statistics, and as upstream phenotype extractors whose learned representations feed conventional association testing. We discuss representative examples of each, before turning to broader genotype-to-phenotype frameworks that bridge sequence-level and population-level modelling.

**SNVformer** [56] demonstrates that transformer attention can scale to GWAS-sized SNV inputs without expanding to the full genome. Each genotyped SNV is mapped to one of 32 tokens encoding allele identity and zygosity, embedded with a sinusoidal positional encoding, and processed by a Transformer encoder using Linformer-style linear-complexity attention [66]. A classification head reads the [CLS] token to perform binary phenotype prediction. The published evaluation predicts gout from *∼*66 000 UK Biobank SNVs (filtered by minor allele frequency and a urate-association screen) on a class-balanced test set of about 4700 individuals, with age, sex, and BMI included as additional inputs, reaching AUROC = 0.83.

SNVformer is a phenotype classifier rather than a variant-discovery method: it does not perform association testing, does not produce per-variant *P*-values or effect-size estimates, and does not directly evaluate epistasis, dominance, or linkage disequilibrium structure. The motivation for capturing non-additive effects is presented in the original paper as a direction for future work rather than as an evaluated capability, with the authors indicating intent to apply attention-based attribution methods in subsequent work to identify contributing SNVs and combinations.

**InsightGWAS (migraine)** [67] introduces a transformer-based variant-prioritization model that operates on GWAS summary statistics rather than on individual genotypes. Each variant is represented by a feature vector combining its summary statistics (effect size, *P*-value, allele frequency, sample size) with multi-omics functional annotations including eQTLs, mQTLs, chromatin accessibility, and conservation scores. A transformer encoder applies self-attention across these features to predict whether a variant is genuinely trait-associated, and the authors explicitly position the model as a post-GWAS prioritization tool that complements rather than replaces conventional association testing.

The training strategy uses transfer learning. The model is first pre-trained on major depressive disorder (MDD) GWAS data using a two-step labelling scheme: positive and negative labels are taken from a well-powered MDD GWAS at the genome-wide significance threshold, while input features are taken from a smaller, lower-powered MDD GWAS, forcing the model to learn the patterns that distinguish true associations under limited statistical power. The pre-trained model is then fine-tuned on the target trait, leveraging genetic correlation between MDD and the target.

Applied to migraine GWAS comprising 53 109 cases and 230 876 controls, InsightGWAS reported 293 previously unreported candidate loci, influencing genes including *CACNA1D, HTR3C*, and *NLGN1*. Two loci, *rs4320030* in *SCN11A* and *rs5763529* in *HORMAD2*, were validated in independent sequencing data. The deep learning model was complemented by cross-trait meta-analyses (MTAG and conditional FDR) to identify pleiotropic loci shared between migraine and MDD, with enrichment analyses uncovering pathways including nitrogen compound metabolism and cation binding.

**InsightGWAS (GERD)** [68] applied the InsightGWAS framework to gastroesophageal reflux disease (GERD) GWAS data, again using MDD as the source trait for pre-training. The architecture and two-step labelling scheme follow the migraine application, with the choice of MDD motivated by the genetic correlation between MDD and GERD ($r_{g} = 0.52$). InsightGWAS recovered known loci and prioritized 209 novel candidate loci enriched in neurogenic, neuromuscular, and epithelial pathways, with enrichment analyses suggesting roles for synaptic transmission and cadherin-mediated signalling. Together with the migraine application, this demonstrates that the InsightGWAS framework generalizes across trait pairs sharing genetic architecture with MDD.

**Vision Transformer Autoencoders for Imaging GWAS** represent a complementary route that brings transformers into GWAS without modifying the association test itself, using a transformer to construct better phenotypes for it. Islam *et al*. [69] train a vision transformer (ViT) autoencoder on UK Biobank T1-weighted brain MRI scans, deriving a 128D latent representation per individual through patch-based self-attention followed by average pooling. These latent dimensions are treated as image-derived phenotypes and used as the trait input for conventional univariate GWAS, run on 22 250 subjects with the standard battery of covariates and a Bonferroni-adjusted significance threshold of $5 \times 10^{-8} / 128$.

Compared with a CNN-autoencoder baseline (UDIP) on the same imaging cohort, the ViT-derived phenotypes yielded 10 additional loci, three of which had no prior association with brain structure in the GWAS Catalog. Interpretation of the model showed that attention concentrated sparsely on a small subset of patches and captured non-local patterns such as inter-hemispheric symmetry, which the authors attribute to the ViT inductive bias relative to convolutional baselines. This work illustrates a third niche for transformers in GWAS: as unsupervised phenotype extractors whose richer representations feed into the standard statistical pipeline, with the discovery gain attributable to the phenotype rather than to a replacement of the association model.

Although the models above operate on reference sequence, the following models predict individual-level phenotype from personal genotypes, beginning with personalized gene-expression prediction. **VariantFormer** [70] extends genomic foundation modelling towards personalized gene expression prediction, addressing a limitation of earlier sequence-based models that operate on reference genomes alone. The architecture is a 1.2 billion parameter two-stage hierarchical transformer that processes diploid personal genomes. An epigenetics modulator first encodes long-range cis-regulatory elements, after which gene-level transformer layers attend to these representations through cross-attention to capture enhancer-promoter interactions and distal regulatory effects.

Trained on 21 004 paired whole-genome and bulk RNA-seq samples from 2330 donors across the Genotype-Tissue Expression project (GTEx), 1000 Genomes project, Alzheimer’s Disease Neuroimaging Initiative (ADNI), and Encyclopedia of DNA Elements project (ENCODE), VariantFormer improves eQTL effect size estimation for lower-frequency and ancestry-specific variants, addressing a known limitation of conventional approaches in underrepresented populations. Applications to Alzheimer’s disease show that learned gene embeddings prioritize likely causal genes, and *in silico* mutagenesis of APOE alleles recovers known risk-modifying effects, bridging sequence-level modelling with individual-level phenotypic variation.

**Phenformer** [71] takes an end-to-end interpretable approach to genotype-to-phenotype prediction at a multi-megabase scale, processing up to 88 megabases of genome sequence per individual, roughly an order of magnitude larger than prior genetic language models. The architecture follows the molecular biology cascade of sequence to cell context to expression to phenotype, through cell-type-specific expression. A pretrained sequence-to-expression backbone based on Enformer [72] first transforms variable numbers of 196-kilobase windows centred on transcription start sites into sequence embeddings. These embeddings are then aggregated by an expression-to-phenotype core of transformer encoder layers with pooling by multihead attention, producing individual-level phenotype risk predictions. Applied to six common diseases in UK Biobank, it outperformed PRS baselines, illustrating transformers can support functionally interpretable genotype-phenotype modelling.

**COMICAL** (Contrastive Multi-Omics Association Learning) [73] frames genotype-phenotype discovery as a multimodal alignment problem rather than a sequence-modelling task. Adapting the CLIP contrastive architecture from vision-language modelling, COMICAL uses two transformer encoders with modality-specific tokenizers to jointly embed SNP genotypes and brain imaging-derived phenotypes (IDPs) into a shared latent space. A contrastive loss aligns representations of paired SNPs and IDPs while separating unrelated pairs, enabling the model to identify many-to-many associations through cross-modal attention. Evaluated on UK Biobank across 10 neurological disorders including Alzheimer’s, COMICAL recovered known SNP-IDP associations with high accuracy, validated against independent GWAS summary statistics, and demonstrated cross-disorder generalization. The approach also yields a multi-omics risk score that correlates with established PRS, illustrating how transformer-based contrastive learning can complement conventional GWAS.

Despite these developments, transformer methods have not displaced conventional GWAS, and it is worth stating why. Standard mixed-model association testing is fast, statistically calibrated, and interpretable at the level of the individual variant: it returns an effect size and a *P*-value per SNP, controls for relatedness and population structure, and rests on an additive model that captures the majority of common-variant heritability for most traits. Transformer approaches offer none of these guarantees directly, they require large labelled cohorts, do not natively produce calibrated per-variant inference, and are harder to interpret so they compete poorly on the task classical methods already perform well. Their advantages lie elsewhere: in modelling non-additive and long-range effects that additive models omit, in learning phenotype representations richer than hand-defined traits (the vision-transformer autoencoder route), and in aligning genotype with other modalities (the contrastive route). This explains the pattern seen across the models reviewed here: the productive applications augment a specific stage of the existing pipeline, phenotype construction, variant prioritization, or genotype-to-phenotype prediction, rather than replacing the association test itself. The remaining methodological challenges for wider adoption are correspondingly specific: calibrated uncertainty quantification, per-variant interpretability that supports biological follow-up, and evaluation stratified by ancestry and linkage-disequilibrium structure rather than on pooled test sets.

Across these examples, transformers intersect with GWAS at three different stages of the pipeline. SNVformer operates on raw genotypes for phenotype classification, InsightGWAS operates on summary statistics for variant prioritization, and the ViT autoencoder operates on imaging data to construct learned phenotypes for downstream testing. Phenformer and COMICAL instead operate end-to-end across genotype and phenotype representations. The diversity is informative: transformer-based GWAS is not a settled methodology, and the most productive applications so far have augmented specific stages of the existing GWAS workflow rather than displaced it.

#### Biodiversity applications

By biodiversity applications we mean the use of transformer models on standardized genetic markers, principally DNA barcodes (short conserved marker regions such as the *mitochondrial COI* gene) to identify organisms to species level, place them taxonomically, and support large-scale biodiversity assessment. We treat biodiversity as a focal subfield because it is the most direct test of whether representations learned on human and model-organism genomes transfer to the far larger and less studied space of non-model taxa; success or failure here bears directly on the generalization analysis of Section Cross-population and cross-species generalization.

**BarcodeBERT** [74] is the principal example. It adapts the BERT framework to DNA-barcode analysis for species identification, demonstrating that transformer modelling extends beyond human genomics to ecological and evolutionary questions. Methodologically it follows the foundation-model recipe: barcode sequences are segmented into non-overlapping k-mer tokens, a model is pre-trained on a large unlabelled barcode corpus with masked language modelling, and the pre-trained backbone is then fine-tuned on labelled data for specific tasks. BarcodeBERT is therefore a foundation model by the criterion of Section Transformer models for genomic analysis, and is listed as such in Table S1; we discuss it here, within the biodiversity subfield, because that is where its methodological significance lies. Reported evaluations span classifying specimens to species level, recovering evolutionary relationships that align with established taxonomy, and supporting the identification of cryptic species and rapid biodiversity assessment from environmental DNA.

BarcodeBERT demonstrates that self-supervised pre-training transfers to non-model taxa, but it also exposes the ceiling of that transfer. Because it is trained on a geographically restricted reference library (Canadian invertebrates), it is the clearest case in this review of a training corpus narrowing the effective generalization surface, a point we return to in Section Cross-population and cross-species generalization.

Across the foundation models of Section Genomic foundation models and the specialized applications of Section Specialized transformer applications, the diversity of architectural choices, training objectives, and target tasks raises the question of how meaningfully these models can be compared. Section Comparative analysis across models turns to that question along three axes: context length and granularity, training paradigm, and cross-population and cross-species generalization.

## Comparative analysis across models

We compare the reviewed models along three axes that matter most for interpreting current capabilities and limitations: context length versus granularity, training paradigm and transferability, and cross-population and cross-species generalization. The coverage of this review relative to prior surveys is given in Table 1; a consolidated comparison of all reviewed models, including their principal strengths and limitations, is given in Table 2; and the detailed per-category specifications (pre-training objective, key innovations, training corpora, and availability) are provided in supplementary material (Tables S1 and S2). Each axis below is supported by a summary of computational requirements (Table 3) and by a dedicated table (Tables 4 to 6), so that the narrative interprets the tables rather than carrying the comparison alone.

**Table 1:** Coverage of this review relative to recent surveys of transformer and language-model approaches in genomics.

Review	Seq. foundation models	Imputation	GWAS pipeline	Genotype to phenotype	Biodiversity	Compute	Population/ancestry
Zhang *et al*. [40]	✓	–	–	–	–	P	–
Consens *et al*. [41]	✓	–	–	P	–	P	–
Benegas *et al*. [42]	✓	–	–	P	–	P	–
Ghosh *et al*. [43]	✓	–	–	–	–	–	–
Choi & Lee [44]	✓	–	–	–	–	–	–
Shu *et al*. [45]	✓	P	–	P	–	P	–
**This review**	✓	✓	✓	✓	✓	✓	✓

✓, substantive treatment; P, partial or brief; –, not covered.

**Table 2:** Consolidated comparison of the transformer and related genome models reviewed. Input length is as reported by the source; strengths and limitations are our assessment. Tokenization is given in Table 4 and detailed training corpora and availability in Supplementary Material.

Model	Architecture	Input length	Primary task(s)	Key strength	Key limitation
DNABERT [30]	Encoder (BERT)	512 bp	Promoter, TFBS, splice	First genomic BERT; interpretable	Short context; k-mer information leakage
DNABERT-2 [31]	Encoder (BERT)	*≈* 10 kb	GUE (28 tasks), multi-species	Efficient (ALiBi, FlashAttention)	Still kilobase-scale
Nucleotide Transformer [32]	Encoder	*≈* 6 kb	Regulatory, epigenetic, variant effect	Multi-species; 50M–2.5B variants	Fixed-length chunks; uneven gains
GENA-LM [33]	Encoder (full/sparse/recur.)	4.5/36 kb	Regulatory, splice, chromatin	Sparse/recurrent long context	Multi-species gains inconsistent
HyenaDNA [46]	Decoder (Hyena conv.)	*≤* 1 Mb	Regulatory benchmarks	Single-base resolution at long context	Not bi-directional
Caduceus [62]	BiMamba SSM	*≤* 131 kb	Variant effect, genomic benchmarks	RC-equivariant; beats 10*×* larger models	Newer; narrower task coverage
Evo [64]/Evo 2 [65]	StripedHyena (v1/v2)	131 kb/1 Mb	Variant effect, sequence design	Genome-scale context; cross-domain	Extreme compute; access-limited
BarcodeBERT [74]	Encoder (BERT)	512 bp	Species ID, taxonomy, eDNA	Transfer to non-model taxa	Geographically restricted corpus
VariantFormer [70]	Two-stage hierarchical	Personal diploid genome	personalized expression, eQTL	Diploid, ancestry-aware eQTL	1.2B params; supervised only
Phenformer [71]	Enformer + transformer	*≤* 88 Mb/indiv.	Disease risk (six traits)	Multi-Mb; interpretable cascade	Backbone-dependent
COMICAL [73]	Dual-encoder (CLIP)	Disease-specific sets	SNP–IDP association (10 disorders)	Cross-modal, many-to-many	Recovers, not replaces, GWAS
SNVformer [56]	Encoder (Linformer)	*≈* 66k SNVs	Gout prediction (AUROC 0.83)	Linear attention at GWAS scale	Classifier, not discovery
InsightGWAS (migraine [67]/GERD [68])	Encoder	Per-variant	Variant prioritization	Transfer across correlated traits	Post-hoc; depends on summary-stat quality
ViT-AE GWAS [69]	ViT autoencoder	Brain MRI	Imaging phenotypes *→* GWAS	+10 loci vs CNN-AE baseline	Phenotype-only contribution
HLARIMNT [54]	Encoder	*≈* 500k SNPs	HLA imputation	Rare-allele gains	HLA-specific
STICI [55]	CNN + transformer	Variable (chunked)	Genotype imputation (SNV/SV)	Reference-free; multi-allelic and SV	Under-rep. ancestries untested

**Table 3:** Computational requirements of representative models, compiled from the primary publications. The uneven reporting is itself a finding (see Sections 5 and 5): the field does not yet report computational cost in a comparable way.

Model	Parameters	Training hardware	Training time/schedule	Public?
DNABERT [30]	*≈* 86–89 M (*k* = 3–6)	8 *×* NVIDIA 2080 Ti	*≈* 25 days	Yes
DNABERT-2 [31]	117 M	8 *×* NVIDIA 2080 Ti	*≈* 14 days	Yes
Nucleotide Transformer [32]	50 M–2.5 B (largest 2.5 B)	2.5 B: 128 *×* NVIDIA A100 across 16 nodes	28 days (2.5 B); *≈*1 day (500 M) on 8 A100	Yes
GENA-LM	110 M/336 M (BigBird 110 M variants)	8 or 16 *×* NVIDIA A100	1–2 M steps, batch 256	Yes
HyenaDNA [46]	1.6 M–13.1 M (long-context commonly 6.6 M)	1 *×* NVIDIA A100 40 GB (benchmark config.)	*≈* 80 min (benchmarked pre-training config.)	Yes
Caduceus [62]	3.9 M (131-kb benchmark config.; larger checkpoints exist)	3090, A5000, A6000, V100, A100 GPUs	NR	Yes
Evo [64]	7 B	Stage 1: 64 *×* NVIDIA H100; Stage 2: 128 *×* NVIDIA A100	NR	Yes
Evo 2 [65]	7 B/40 B	NVIDIA DGX Cloud (AWS), >2000 H100	NR	Yes
VariantFormer [70]	1.2 B	64 *×* NVIDIA H100	NR	Yes
Phenformer [71]	NR	Distributed NVIDIA A100 DGX and Tesla V100 HGX	12,500 GPU-hours	Restricted
SNVformer [56]	NR	1 *×* NVIDIA Quadro RTX 8000 (48 GB)	NR	Yes
BarcodeBERT [74]	29.1 M	4 *×* NVIDIA V100	*≈* 36 h	Yes

NR, not reported; *≈*, approximate or architecture-scale figure. Memory and inference requirements are omitted because none of the reviewed publications report them. Models not listed (InsightGWAS, ViT-AE GWAS, HLARIMNT, STICI, COMICAL) do not report parameter counts or training details.

**Table 4:** Context length versus granularity for the sequence-level models. Maximum context and resolution are as reported by the source publications.

Model	Resolution/tokenization	Max. context	Attention/operator
DNABERT [30]	Overlapping 6-mer	512 bp	Full attention
DNABERT-2 [31]	BPE	*≈* 10 kb	Full attention (FlashAttn, ALiBi)
Nucleotide Transformer [32]	Non-overlapping 6-mer	*≈* 6 kb	Full attention
GENA-LM [33]	BPE	4.5 kb/36 kb	Full/BigBird sparse/recurrent
HyenaDNA [46]	Single-nucleotide	*≤* 1 Mb	Long convolution (Hyena)
Caduceus [62]	Single-nucleotide	*≤* 131 kb	State-space (BiMamba, RC-equiv.)
Evo [64]/Evo 2 [65]	Single-nucleotide	131 kb/1 Mb	StripedHyena (conv. + attention)
Phenformer [71]	Enformer embeddings	*≤* 88 Mb	Hybrid (Enformer + transformer)

**Table 5:** Training paradigm and transferability. Models fall into three groups that are rarely evaluated on common tasks, which is the principal obstacle to fair head-to-head comparison.

Group	Models	Pre-training signal	Transfer breadth/comparators
Self-supervised foundation	DNABERT family, Nucleotide Transformer, GENA-LM, HyenaDNA, Caduceus, Evo/Evo 2, BarcodeBERT	Masked or autoregressive sequence modelling	Broad; many downstream tasks; benchmarked against other foundation models (e.g. GUE)
Supervised pre-training	VariantFormer, Phenformer (Enformer backbone)	Supervised on the target signal	Narrow; strong on target task; benchmarked against task baselines and PRS
Single-application	HLARIMNT, STICI, SNVformer, InsightGWAS, ViT-AE GWAS, COMICAL	Task-specific (some self-supervised masking)	None intended; benchmarked against classical baselines

**Table 6:** Cross-population and cross-species generalization. “Under-rep. evaluated?” records whether the source publication explicitly benchmarks underrepresented populations.

Model	Training-corpus breadth	Under-rep. evaluated?	Observed generalization limit
Nucleotide Transformer [32]/GENA-LM [33]	Human + multi-species	No	Multi-species training does not consistently help human tasks
DNABERT-2 [31]	Human + multi-species	No	Cross-species gains are task-dependent
HLARIMNT [54]	Pan-Asian + T1DGC panels	No	Ancestry robustness untested
STICI [55]	Human + yeast, rat, chicken	No	Underrepresented human ancestries untested
BarcodeBERT [74]	Canadian invertebrates	No	Geographic specialization limits transfer
VariantFormer [70]	GTEx, 1000G/MAGE, ADNI, ENCODE	Partial	Improves lower-frequency and ancestry-specific eQTL, but corpus remains European-weighted

### Context length versus granularity

There is a fundamental trade-off between the resolution at which a model represents sequence and the context length it can process (Table 4). DNABERT operates at high resolution (overlapping 6-mers) but is capped at 512 bp, sufficient for promoter and splice-site analysis but unable to model enhancer–promoter interactions. DNABERT-2 and the Nucleotide Transformer extend the range through BPE and longer input windows but remain in the kilobase regime. GENA-LM pushes to 36 kb via BigBird-style sparse attention, and Phenformer reaches multi-megabase scale by stacking an Enformer backbone under a transformer pooling head. The trade-off is not monotonic: GENA-LM shows that context beyond 16 kb measurably improves promoter prediction (F1 94.6% versus 93.7% at 2 kb), yet DNABERT-2 still wins on most short-context GUE benchmarks. The choice of context length should therefore be matched to the spatial scale of the biological signal. The long-context and SSMs of Section Beyond attention: long-context and state-space models push this axis to its limit, with Evo 2 reaching one megabase, but the same caveat applies with greater force: the value of megabase context is established for only a handful of tasks, and demonstrating that long-range signal is genuinely used, rather than merely accessible, is now the central evaluation problem for this class of model.

### Training paradigms and transferability

Self-supervised foundation models (DNABERT family, Nucleotide Transformer, GENA-LM, BarcodeBERT) are pre-trained with masked language modelling and fine-tuned to many tasks; their value is breadth of transfer rather than peak performance. Models with supervised pre-training (VariantFormer, Phenformer, Enformer) achieve stronger results on the target task but transfer less readily. Single-application models (HLARIMNT, STICI, SNVformer) are not designed to generalize beyond their defined task, and their reported comparisons are against classical baselines rather than with the foundation models. This stratification, summarized in Table 5, is the principal reason fair cross-model comparison is difficult: the three groups are almost never evaluated on the same tasks.

### Cross-population and cross-species generalization

Most models are trained predominantly on European-ancestry or single-species data (Table 6). Multi-species training does not consistently improve human-task performance in the GENA-LM family, indicating that data diversity alone is insufficient. Neither HLARIMNT nor STICI specifically evaluates underrepresented populations, leaving cross-population robustness empirically unresolved. BarcodeBERT, trained exclusively on Canadian invertebrates, represents the clearest case of geographic specialization limiting generalization. Across all models, the effective generalization surface is narrower than the training-corpus breadth suggests, a practical bottleneck for clinical and conservation deployment (Section Data quality and population representation).

A final observation is that the models reviewed target different pipeline stages (phenotype classification, post-GWAS prioritization, upstream phenotype extraction, multimodal alignment, sequence imputation) and report incommensurable metrics (AUROC, imputation accuracy, F1, novel-locus counts). The consequences of this heterogeneity for benchmarking and reproducibility are taken up in Section Benchmarking and reproducibility; here we note only that until cross-cutting benchmarks evaluate the same architecture across multiple pipeline stages, comparative analysis must remain in part qualitative.

## Broader challenges and limitations in genomic transformer research

Several fundamental challenges limit widespread adoption and optimal performance, spanning computational, methodological, and ethical dimensions, requiring systematic investigation and coordinated research efforts.

### Computational scalability

Standard self-attention scales quadratically with sequence length [25], creating bottlenecks for genome-scale inputs that span millions of base pairs. Training DNABERT-2 across multiple species required a distributed high-end GPU cluster [31], a resource unavailable to many academic groups. Existing mitigations like sparse attention (BigBird in GENA-LM [33]), linear attention [75], and segmentation strategies [60] partially address this but lack genomic-specific optimization (Section Computational efficiency and scalability). The long-context and SSMs of Section Beyond attention: long-context and state-space models illustrate both the mitigation and the residual problem. Sparse attention, linear attention, long convolutions, and state-space operators reduce the scaling of context length from quadratic towards linear, yet the largest long-context models, such as Evo 2 at 40 billion parameters and one-megabase context, require industrial training infrastructure beyond the reach of most individual laboratories. The bottleneck therefore shifts from algorithmic complexity to capital cost, and with it to the question of who is able to build such models.

### Data quality and population representation

Transformer pre-training corpora are drawn almost exclusively from European-ancestry reference genomes, the 1000 Genomes Project, and a small set of model organisms. Martin *et al*. [76] demonstrated that polygenic risk scores from European-ancestry data show significantly reduced accuracy in non-European populations, a limitation that genomic transformers inherit at the representation level. These representation-level biases are not corrected by fine-tuning on diverse labels, because they are encoded during pre-training; a model pre-trained predominantly on European-ancestry references inherits that population’s linkage and haplotype structure regardless of the downstream task. Addressing this requires inclusive dataset development, algorithmic fairness evaluation, and federated learning approaches that allow diverse cohorts to contribute without centralizing sensitive data, with ancestry-stratified evaluation treated as a default rather than an afterthought.

### Interpretability and biological validation

Transformers are often credited with interpretability through their attention weights, but this advantage is narrower than it appears, and a critical review must distinguish several distinct approaches. Attention-based explanations visualize which positions a head attends to; they are cheap and intuitive but do not establish causality, can co-vary with spurious correlations, and, as shown in the natural-language literature, need not correspond to feature importance at all [77–79]. *Gradient- and attribution-based* methods (saliency, integrated gradients, DeepLIFT-style attributions) assign importance to input bases and are more faithful than raw attention, but remain sensitive to baseline choice and to correlated inputs, which are pervasive in DNA because of linkage disequilibrium. *Perturbation-based* methods, chief among them *in silico* mutagenesis, measure the change in prediction when each base is altered and align most closely with the experimental notion of a variant effect, at the cost of being computationally expensive at genome scale. *Mechanistic interpretability*, which decomposes the network into internal features and circuits, has begun to be applied to genomic models, including feature-extraction analyses of the Evo family [65], and offers the prospect of explanations at the level of learned regulatory concepts rather than raw positions, though the work is early. The common limitation across all four is that a computational attribution, however faithful to the model, is a hypothesis about biology, not evidence of it. Experimental validation, whether by CRISPR perturbation, reporter assays, or eQTL concordance, remains necessary before biological or clinical claims, as the CRISPR follow-up required to confirm DNABERT-2 predictions illustrates [31]. Progress therefore depends less on new visualization methods than on coupling attribution to mechanistic validation and to causal-inference frameworks.

### Ethical considerations and clinical translation

Genomic transformers may memorize individual-level haplotype patterns from the pre-training corpus, with adversarial probing able to recover training fragments [80]. Traditional anonymization is insufficient, since individuals can be reidentified from aggregated genomic datasets [81]. Expanding capabilities to derive novel phenotypic information also challenge existing informed-consent frameworks [82, 83]. Federated learning with differential privacy and secure aggregation offers a technically mature response [84, 85], but open challenges remain in handling statistical heterogeneity across sites, defending against gradient-based reconstruction attacks, and reconciling cross-border training with health data regulations. Beyond privacy and consent, deployment raises questions of robustness and openness. Models vary widely in adherence to FAIR and open-source practice, i.e. in the release of weights, code, training data, and evaluation protocols, and this variation, visible in the final column of Table 3, directly determines whether independent groups can audit, reproduce, or safely deploy a model. Robustness to distribution shift across ancestries, sequencing platforms, and cohorts is rarely characterized, yet it is precisely what clinical deployment requires. We therefore regard ancestry-stratified, platform-aware robustness evaluation and open release as deployment prerequisites, not optional additions.

### Benchmarking and reproducibility

A recurring obstacle across the models reviewed here is the absence of standardized evaluation. Benchmarks are heterogeneous: some foundation models report on the GUE suite, others on bespoke task collections, and the specialized applications are compared almost exclusively against classical baselines rather than against one another or against foundation models. The reported metrics are themselves incomparable, spanning AUROC for phenotype classifiers, imputation accuracy for imputation models, F1 for regulatory tasks, and novel-locus counts for GWAS applications, so that “state-of-the-art” claims cannot be aggregated across the field. Reproducibility is further limited by inconsistent release of code, weights, and, as Table 5 shows, the computational details needed to reproduce or even fairly compare training. Two changes would do more for the field than further architectural innovation: cross-cutting benchmarks that evaluate a single architecture across multiple pipeline stages (sequence annotation, imputation, association, phenotype prediction), and a reporting standard for model size, training cost, and data provenance. Until these exist, the comparative analysis of Section Comparative analysis across models must remain in part qualitative, not for lack of models but for lack of common ground on which to compare them.

## Future directions and research opportunities

The analysis reveals that while transformers have demonstrated genomic sequence analysis capabilities, significant opportunities remain to advance their impact on functional genomics. This section outlines our vision for addressing Section Broader challenges and limitations in genomic transformer research ’s identified limitations, organized into four key areas.

### Multi-modal data integration

Current genomic transformers process single data modalities, limiting their ability to capture interactions between genetic variation, epigenetic modifications, and phenotypic outcomes. Unified multi-omics architectures that simultaneously process genomic sequences, transcriptomic profiles, epigenomic modifications, and proteomic data within shared latent spaces, using cross-modal attention to model biological relationships explicitly, would reveal systems-level patterns undetectable in single-modality analyses. Temporal multimodal modelling could further capture how genomic variants influence longitudinal changes in gene expression and clinical phenotypes, with applications to disease progression and treatment timing.

### Biological knowledge integration

Current models learn entirely from data, without incorporating knowledge of regulatory networks, pathway interactions, or evolutionary constraints. Attention mechanisms could be redesigned to integrate prior knowledge: initializing or constraining attention weights based on known regulatory interactions, or structuring sparsity to preferentially connect enhancers with target promoters and co-regulated genes. Pre-training tasks could explicitly leverage biology: predicting evolutionary conservation, reconstructing disrupted pathway relationships, enabling models to learn causal mechanisms rather than correlations. Architectures that mirror the genomic hierarchy (nucleotides *→* motifs *→* regulatory elements *→* networks *→* phenotypes) would improve sample efficiency and interpretability.

### Computational efficiency and scalability

The quadratic complexity of standard self-attention limits most current models to sequences under 10 kb, yet many biological phenomena span hundreds of kilobases to megabases. Genomically informed attention patterns that are structured to connect promoters with regulatory regions, exons within genes, or regions within topologically associating domains could reduce computational requirements while preserving biological relevance. Hybrid architectures combining transformer attention with recurrent memory mechanisms (building on Transformer-XL [86] and Compressive Transformers [87]) could enable chromosome-scale processing on accessible hardware.

### Clinical translation and validation

Three properties of genomic transformers create translation challenges beyond conventional GWAS pipelines. First, fine-tuned models inherit representation gaps from their pre-training corpus: a transformer pre-trained on European-ancestry genomes will underperform on diverse populations even when fine-tuned on diverse labels. Validation must therefore report performance stratified by ancestry and LD structure, not only on held-out test sets. Second, attention-based interpretability must be paired with mechanistic validation (CRISPR perturbation, reporter assays, eQTL evidence) before clinical action; attention alone does not establish causality (Section Interpretability and biological validation). Third, current regulatory frameworks assume a model is trained and validated as a unit, but fine-tuned genomic transformers share most parameters with a foundation model updated on a separate schedule. Whether validation must be rerun after backbone updates, and how to monitor performance drift across ancestrally heterogeneous deployment populations, remain open questions not addressed by existing AI-in-medicine guidance [88]). Embedding ancestry-stratified evaluation and mechanistic validation into benchmarking workflows would do more for clinical translation than further architectural innovation.

The directions above address distinct limitations, but they share a common prerequisite: infrastructure for evaluation and comparison that the field does not yet have. Looking beyond incremental gains, we regard five problems as decisive for whether transformer genomics becomes a settled methodology. First, **cross-pipeline benchmarks**: the field cannot presently compare a model across annotation, imputation, association, and phenotype prediction, and building such benchmarks is a precondition for meaningful progress (Section Benchmarking and reproducibility). Second, **ancestry- and LD-stratified validation** reported as standard, so that generalization is measured rather than assumed. Third, **genomically structured attention and operators**: sparsity and state-space designs that connect promoters to their regulatory regions or exons within genes, rather than generic efficiency mechanisms, so that long context carries biological signal (Section Computational efficiency and scalability). Fourth, **standardized reporting of compute and data provenance**, without which reproducibility and equitable access are unattainable. Fifth, **integration of long-context state-space backbones** (HyenaDNA, Caduceus, Evo 2) with the population- and phenotype-level applications that have so far relied on shorter transformers, closing the gap between sequence-level and population-level modelling that motivates this review. These are the directions on which the field’s trajectory, more than any single architecture, will depend.

## Conclusion

This review examined the rapidly evolving landscape of transformer-based models for genomic data analysis, spanning sequence-level foundation models, population-level applications in imputation and GWAS, and biodiversity research.

Across these areas, transformers have moved from architectural exploration to delivering measurable advances on specific tasks: foundation models now provide strong general-purpose sequence representations, GWAS-pipeline applications have begun to augment specific stages of the conventional workflow, and imputation and biodiversity transformers extend coverage to traditionally difficult regions and to non-human species. At the same time, the field’s benchmarks are heterogeneous, the models target different pipeline stages, and direct head-to-head comparison is often not possible. The comparative reading offered in Section Comparative analysis across models highlights three axes (context length, training paradigm, generalization) along which qualitative comparison is currently meaningful.

**Looking forward**, the most consequential research directions are those that move beyond incremental performance gains. As set out in Section Future directions and research opportunities, these turn less on new architectures than on the benchmarks, stratified validation, standardized reporting, and biologically structured designs needed to make existing models comparable and trustworthy. Equity, privacy, and ancestrally inclusive evaluation are not optional addenda but conditions on whether these methods will be useful outside their training distributions.

Whether transformer-based models become a settled methodology in genomics or remain a heterogeneous family of specialized tools will depend less on architectural innovation and more on the field’s ability to build the benchmarks, datasets, and validation pipelines that turn promising architectures into trustworthy methods.

**Key Points**

- Transformer models in genomics have a variety of application areas, including imputation, GWAS pipelines, end-to-end genetic prediction, biodiversity, and sequence annotation.
- Transformer-based genomics is not yet a single mature methodology but a rapidly evolving collection of approaches that enter the genomic analysis pipeline at different stages.
- Their limitations and challenges include methodological trade-offs, including context length, granularity, transferability, interpretability, scalability, and cross-population and task generalization.

## Supplementary Material

GWAS_deeplearning_transformer_review_BIB_supp_bbag628

## Data Availability

Data used in this article were retrieved from publicly accessible journals and preprint archives.
