# Supplementary material for: Transformer models in genomics: from sequence analysis to population genetics and biodiversity
Source: Brief Bioinform. 2026 Nov 16;27(6):bbag628. doi: 10.1093/bib/bbag628 (PMC13770719; doi:10.1093/bib/bbag628)
Supplement: Supplementary file 1 [file gwas_deeplearning_transformer_review_bib_supp_bbag628.pdf]

Supplementary Material

Transformer Models in Genomics: From Sequence Analysis to Population Genetics and Biodiversity

Detailed Model Specifications

This supplementary file provides the detailed per-category specifications summarised in the main-text comparison table (Table 2). Models are grouped by the classification criterion of Section 3 of the main text: Table S1 lists the foundation models, pre-trained with self-supervised objectives on large unlabelled corpora and adapted via fine-tuning; Table S2 lists the specialised applications, trained, with or without supervised pre-training, for a single defined task. To avoid duplication with the main-text tables, architecture, tokenisation, and input length are given in the main-text comparison tables (Tables 2 and 4); the tables below give the pre-training objective, key innovations, training corpora, and availability. MLM = masked language modelling; AR = autoregressive; SS = GWAS summary statistics.

Table S1. Genomic foundation models. Re-scoped to training provenance and availability; architecture and tokenisation are in the main-text comparison tables (Tables 2 and 4). Long-context and state-space genome language models (HyenaDNA, Caduceus, Evo/Evo 2) are included as foundation models by the criterion of Section 3.

Model	Pre-training	Key innovations	Training corpora	Public?
DNABERT [30]	MLM	First application of BERT to genomic DNA	Human reference genome (hg38)	Yes
DNABERT-2 [31]	MLM	ALiBi positional encoding, FlashAttention, low-precision LayerNorm, multi-species pre-training	Human and multi-species genomes	Yes
Nucleotide Transformer [32]	MLM	Multi-species pre-training; 50M–2.5B parameter variants	Human reference (hg38), 1000 Genomes, multi-species dataset	Yes
GENA-LM [33]	MLM	Full / sparse / recurrent attention variants; sparse attention adapted from BigBird	Human T2T v2 assembly, multi-species, 1000 Genomes	Yes
HyenaDNA [46]	AR	Implicit long convolutions; single-nucleotide resolution to 1 Mb context	Human reference genome	Yes
Caduceus [62]	Self-sup.	Bi-directional, reverse-complement-equivariant state-space (Mamba) model	Human reference genome	Yes
Evo / Evo 2 [64, 65]	AR	StripedHyena / StripedHyena 2; 7B/40B params; genome-scale context across domains of life	OpenGenome / OpenGenome2 (≈9.3 Tbp)	Yes
BarcodeBERT [74]	MLM	Domain-specific pre-training on DNA barcodes for biodiversity classification	Canadian invertebrate barcode reference library	Yes

Table S2. Transformer models for specialised genomic applications. Re-scoped to training provenance and availability; architecture, tokenisation, and input size are in the main-text comparison tables (Tables 2 and 4). SS = GWAS summary statistics; PRS = polygenic risk score; IDP = image-derived phenotype.

Model	Training objective	Key innovations	Training corpora	Public?
Gene expression and genotype-to-phenotype modelling				
VariantFormer [70]	Supervised (paired WGS + RNA-seq)	1.2B-parameter cross-attention between cis-regulatory and gene-level layers; diploid-aware expression prediction	GTEx, MAGE, ADNI, ENCODE	Yes / re-stricted
Phenformer [71]	End-to-end supervised on phenotype	Multi-megabase scale; sequence → cell context → expression → phenotype cascade; PMA pooling	UK Biobank	Restricted
COMICAL [73]	Contrastive (multimodal)	Cross-modal attention; many-to-many SNP–phenotype associations; multi-omics PRS-correlated risk score	UK Biobank, ENIGMA summary statistics	Yes / re-stricted
GWAS-pipeline applications				
SNVformer [56]	Supervised (binary phenotype classification)	Linear-complexity attention scaled to GWAS-sized inputs; phenotype classifier, not a variant-discovery method	UK Biobank	Restricted
InsightGWAS (migraine) [67]	Supervised: pre-train on MDD, fine-tune on migraine	Combined with MTAG and conditional-FDR cross-trait analyses; same framework as the GERD application	Migraine and MDD GWAS summary statistics	Yes
InsightGWAS (GERD) [68]	Supervised: pre-train on MDD, fine-tune on GERD	Two-step labelling scheme; post-GWAS variant prioritisation via cross-trait transfer learning	GERD and MDD GWAS summary statistics	Yes
ViT-AE GWAS [69]	Reconstruction (self-supervised)	Learned imaging phenotypes for downstream linear GWAS; sparse attention captures non-local patterns (e.g. inter-hemispheric symmetry)	UK Biobank brain MRI	Restricted
Genotype imputation				
HLARIMNT [54]	Supervised (HLA classification)	Specialised for HLA imputation; handles rare alleles	Pan-Asian and T1DGC reference panels	Some panels public
STICI [55]	Self-supervised masking (reconstruction)	Multi-scale convolutional blocks; reference-free imputation; supports multi-allelic and structural variants	1000 Genomes (HLA, chr-22, SVs), yeast, rat, chicken	Yes

Glossary of Terms

Machine learning and architecture terms

ALiBi (Attention with Linear Biases). A positional encoding scheme that biases attention scores by a distance-dependent linear penalty rather than adding learned or sinusoidal position embeddings. Allows transformers to extrapolate to sequence lengths longer than those seen during training.

Attention head. One parallel sub-component of a multi-head attention layer. Each head independently learns a different set of query, key, and value projections, allowing the model to attend to different aspects of the input simultaneously.

Autoregressive modelling. A self-supervised learning objective in which a model predicts each token in a sequence given only the tokens that precede it. Used in generative language models such as GPT.

Byte Pair Encoding (BPE). A subword tokenization algorithm that iteratively merges the most frequent adjacent token pairs to build a variable-length vocabulary. In genomics, BPE allows recurrent biological motifs to emerge as single tokens without prior biological knowledge.

[CLS] token. A special "classification" token prepended to the input sequence in BERT-style transformer models. Its final-layer embedding serves as a summary representation of the entire input and is fed into a classification head for sequence-level prediction tasks.

Contrastive learning. A self-supervised learning paradigm in which a model is trained to bring representations of related (positive) pairs closer together in latent space while pushing apart unrelated (negative) pairs.

Cross-attention. An attention mechanism in which queries come from one sequence (or modality) and keys/values come from another, enabling information flow between distinct inputs such as sequence and expression representations.

Embedding. A learned dense vector representation of a discrete input element such as a token, k-mer, or SNP.

Fine-tuning. The process of adapting a pre-trained model to a specific downstream task by continuing training on a smaller labelled dataset, typically with a task-specific head.

FlashAttention. An I/O-aware exact attention algorithm that reorganises memory access patterns to reduce GPU memory usage and accelerate transformer training without changing the underlying computation.

Foundation model. A model pre-trained on broad data at scale that can be adapted (via fine-tuning or prompting) to a wide range of downstream tasks. In genomics, foundation models are pre-trained on large unlabelled sequence corpora.

Linformer. A transformer variant that approximates self-attention with linear rather than quadratic complexity in sequence length by projecting keys and values to a lower-dimensional space.

LoRA (Low-Rank Adaptation). A parameter-efficient fine-tuning technique that injects small low-rank update matrices into a frozen pre-trained model, dramatically reducing the number of trainable parameters.

Masked Language Modelling (MLM). A self-supervised learning objective in which a fraction of tokens in the input sequence are randomly masked, and the model is trained to predict their identities from the surrounding bidirectional context. Introduced in BERT and adapted by DNABERT and related genomic models.

Multi-head self-attention. An attention mechanism in which queries, keys, and values are projected into multiple parallel subspaces, attention is computed in each subspace, and the results are concatenated. Allows the model to capture different types of relationships at different positions simultaneously.

Positional encoding. Information added to token embeddings to indicate their position in the sequence, since self-attention is otherwise position-invariant. Encodings may be fixed (sinusoidal), learned, relative, or biased (e.g. ALiBi).

Pre-training. The first stage of transfer learning, in which a model is trained on a large unlabelled or weakly labelled corpus using a self-supervised objective to learn general-purpose representations.

Self-attention. The mechanism by which each position in a sequence computes a weighted combination of representations of all positions, with weights determined by learned similarity between query and key vectors.
Self-supervised learning (SSL). A learning paradigm in which supervisory signals are generated from the input data itself through carefully designed pretext tasks, removing the need for manual labels.
Sparse attention. Any attention pattern in which each token attends to only a subset of other tokens, reducing the quadratic cost of full attention.
Tokenization. The process of dividing a raw input sequence into discrete units (tokens) that serve as the input vocabulary for a model.
Transfer learning. The practice of leveraging knowledge from a model trained on one task or dataset (typically through pre-training) to improve performance on a related downstream task.

Genomics and biology terms
Cis-regulatory element (CRE). A non-coding DNA region (e.g. a promoter or enhancer) that regulates the transcription of nearby genes on the same chromosome.
DNA barcode. A short, standardised genetic marker (typically a region of the mitochondrial *COI* gene in animals or rbcL/matK in plants) used to identify organisms to species level.
Enhancer. A regulatory DNA element, often distal to its target gene, that increases transcription rate when bound by transcription factors.
Environmental DNA (eDNA). Genetic material recovered from environmental samples such as soil, water, or air, used for biodiversity monitoring without direct sampling of organisms.
Epistasis. The phenomenon by which the phenotypic effect of one genetic variant depends on the genotype at one or more other loci.
Expression quantitative trait locus (eQTL). A genetic variant whose genotype is statistically associated with the expression level of a gene.
Gene Ontology (GO). A structured controlled vocabulary describing gene and gene product attributes across biological processes, molecular functions, and cellular components.
Genome-wide association study (GWAS). A study design that tests millions of genetic variants across many individuals to identify those statistically associated with a trait or disease.
Haplotype. A combination of alleles at adjacent loci on the same chromosome that tend to be inherited together.
Hidden Markov Model (HMM). A probabilistic model in which a sequence of observed symbols is generated by an underlying sequence of hidden states with Markov transitions. Profile HMMs are widely used in sequence homology detection.
HLA (Human Leukocyte Antigen). A highly polymorphic gene complex on chromosome 6 encoding cell-surface proteins central to immune recognition; key for transplantation matching and disease association studies.
Imputation (genetic). The statistical inference of unobserved genotypes in a study sample using a reference panel of more densely genotyped individuals, leveraging linkage disequilibrium.
Linkage disequilibrium (LD). The non-random association of alleles at different loci in a population.
Major Histocompatibility Complex (MHC). A region of the genome (containing the HLA genes in humans) encoding proteins essential to immune system function.
Minor allele frequency (MAF). The frequency of the second-most-common allele at a given variant in a population. Variants are commonly stratified into common (MAF $> 5\%$), low-frequency (1–5%), and rare ($< 1\%$) categories.
Motif. A short, recurring sequence pattern, often the binding site of a regulatory protein such as a transcription factor.
Polygenic risk score (PRS). A weighted sum of trait-associated alleles carried by an individual, used to estimate genetic predisposition to a complex trait or disease.
Position weight matrix (PWM). A matrix encoding the probability of observing each nucleotide at each position of a fixed-length sequence motif. Foundational in computational sequence analysis.

Promoter. The DNA region immediately upstream of a gene's transcription start site that recruits transcription machinery to initiate RNA synthesis.
Single nucleotide polymorphism (SNP). A genetic variant at which a single nucleotide differs between individuals in a population.
Single nucleotide variant (SNV). A single-base difference between two genomes; broader than SNP, since SNVs include rare and somatic variants not yet established as polymorphisms.
Topologically associating domain (TAD). A self-interacting genomic region in which DNA sequences contact each other more frequently than they contact sequences outside the region; an organisational unit of 3D chromatin structure.
Transcription factor (TF). A protein that binds specific DNA sequences (motifs) to regulate gene transcription.
Transcription start site (TSS). The position in a gene at which transcription is initiated.
Transcriptome-Wide Association Study (TWAS). A study design that uses genetically predicted gene expression to test for association between gene expression and a trait, integrating eQTL information with GWAS.
